# High throughput analysis reveals dissociable gene expression profiles in two independent neural systems involved in the regulation of social behavior

**DOI:** 10.1186/1471-2202-13-126

**Published:** 2012-10-19

**Authors:** Tyler J Stevenson, Kirstin Replogle, Jenny Drnevich, David F Clayton, Gregory F Ball

**Affiliations:** 1 Department of Psychological and Brain Sciences, Johns Hopkins University, Baltimore, Maryland, USA; 2 Department of Cell and Developmental Biology, University of Illinois, Champaign, Illinois, USA; 3 Institute for Genomic Biology, University of Illinois, Champaign, Illinois, USA; 4 High Performance Biological Computing Program and the Carver Biotechnology Center, University of Illinois at Urbana-Champaign, Urbana, Illinois, USA; 5 Neuroscience Program, University of Illinois, Champaign, Illinois, USA; 6 Institute for Mind and Biology, University of Chicago, Chicago, Il, 60637, USA

**Keywords:** Songbird, Microarray, Plasticity, Reproduction, Starling, POA, HVC, Area X

## Abstract

**Background:**

Production of contextually appropriate social behaviors involves integrated activity across many brain regions. Many songbird species produce complex vocalizations called ‘songs’ that serve to attract potential mates, defend territories, and/or maintain flock cohesion. There are a series of discrete interconnect brain regions that are essential for the successful production of song. The probability and intensity of singing behavior is influenced by the reproductive state. The objectives of this study were to examine the broad changes in gene expression in brain regions that control song production with a brain region that governs the reproductive state.

**Results:**

We show using microarray cDNA analysis that two discrete brain systems that are both involved in governing singing behavior show markedly different gene expression profiles. We found that cortical and basal ganglia-like brain regions that control the socio-motor production of song in birds exhibit a categorical switch in gene expression that was dependent on their reproductive state. This pattern is in stark contrast to the pattern of expression observed in a hypothalamic brain region that governs the neuroendocrine control of reproduction. Subsequent gene ontology analysis revealed marked variation in the functional categories of active genes dependent on reproductive state and anatomical localization. HVC, one cortical-like structure, displayed significant gene expression changes associated with microtubule and neurofilament cytoskeleton organization, MAP kinase activity, and steroid hormone receptor complex activity. The transitions observed in the preoptic area, a nucleus that governs the motivation to engage in singing, exhibited variation in functional categories that included thyroid hormone receptor activity, epigenetic and angiogenetic processes.

**Conclusions:**

These findings highlight the importance of considering the temporal patterns of gene expression across several brain regions when engaging in social behaviors.

## Background

The relationship between genes and social behavior involves complex interactions between an individual and its environment [1,2]. Great strides have been taken to identify a number of genes that underlie naturally occurring behavioral plasticity [1-4]. However the vast majority of studies examining the genetic control of social behavior have focused on specific genes or whole brain analyses [1-4]. Indeed, social behavior requires the interaction of a number of different brain regions within a neural circuit that leads to the production of a given behavior. Seasonally breeding animals exhibit one of the most pronounced patterns of behavioral and brain plasticity known and importantly, these changes in brain and behavior are reversible. By studying the neural control of seasonal social behavior one can gain insight into the fundamental mechanisms of gene, brain and behavior relations.

**Figure 1:**
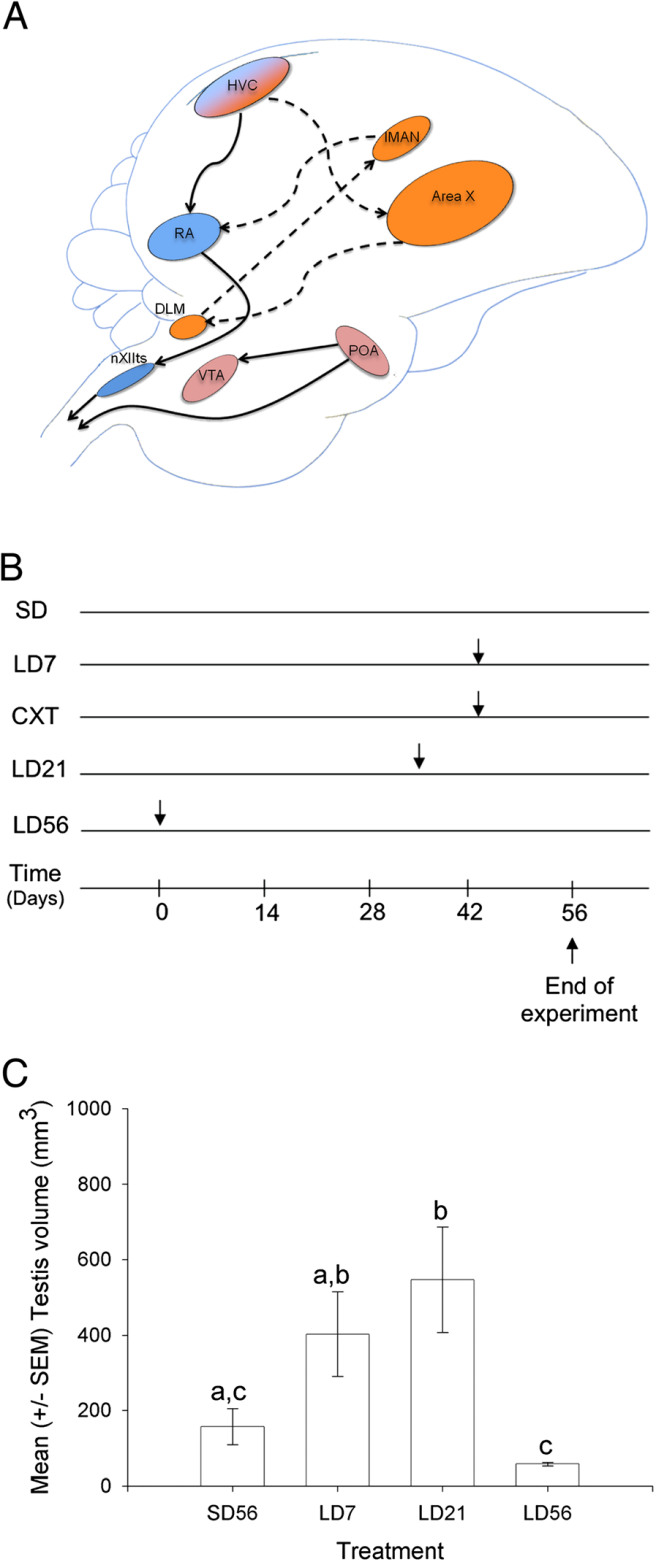
**A) The neural circuitry for song production and the motivation to engage in singing behavior.** The motor pathway for song is demarcated by solid black lines. Song is initiated by motor sequences in HVC neurons that project to the premotor nucleus RA. RA innervates the hindbrain nucleus nXIIts that ultimately controls the vocal organ, the syrinx. The anterior forebrain pathway includes Area X, DLM, lMAN and RA and the circuit is indicated by the dashed black lines. The hypothalamic nucleus, POA projects to the ventral tegmental area (VTA) and the spinal cord. This circuit is essential for the motivation to engage in singing behavior and male copulatory behavior. **B**) A schematic diagram of the experimental treatment groups. Each row represents an experimental treatment group and the horizontal axis represents the experiment timeline measured in the number of days. Arrows indicate the time when starlings were transferred to a long day photoperiod. **C**) The mean (+/− SEM) testicular volume from starlings at the termination of the experiment. The change in volume provides a reliable marker for necessary photoperiodic manipulations of reproductive state. Letters indicate significant differences between treatment groups.

Avian species in the order Passeriformes engage in complex vocal communication (i.e. singing) that is a highly social behavior [5]. Discrete interconnected regions in the passerine brain control the vocal variability and stereotyped motor production required to produce an individual’s song [6,7](Figure 1A). Activity within these regions varies with the social environment to produce the contextually appropriate song [8,9]. HVC (acronym used as a proper name) is a sensorimotor nucleus that governs the motor production of song via the premotor nucleus RA (robust nucleus of the arcopallium) [6,10]. A second afferent projection from HVC innervates the nucleus Area X. This pathway is involved in processing auditory feedback needed to learn and maintain song, inducing vocal variability during song learning and adjusting song quality according to the social context [6,8,11,12]. Together, these brain regions are part of a neural circuit regulating song behavior known as the song control system [5].

Songbirds provide a valuable model to study the neurogenetics of social behavior due to the extensive and reliable changes in song nuclei that govern singing behavior [13,14]. In particular, the European starling (*Sturnus vulgaris*) exhibits marked variability in song control region morphology (e.g., changes in nucleus volume) across the year and these changes are dependent on the reproductive state [15]. European starlings, like many seasonally breeding songbirds, also exhibit marked plasticity in the neuroendocrine control of reproduction [16]. The seasonal change in output signal from the preoptic area (POA) leads to a significant increase in testosterone concentrations in the spring and summer that in turn governs the probability and intensity in which birds will engage in song and the change in singing behavior is also dependent on the social context [14,17]. In addition to regulating the motivation to sing, the seasonal increase in testosterone concentrations is essential for regulating aspects of adult neurogenesis and neural migration, angiogenesis, and synaptogenesis observed in many song control regions in male songbirds, in particular HVC [12,18-20]. The substantial changes in the neural attributes that arise from testosterone action (or the metabolite estrogen) are necessary for the increase in the quality of song produced [21,22].

Here we examined the genetic profile of four brain regions from two independent systems that control a highly complex social behavior (i.e. song) in male European starlings. Using microarray cDNAs designed for use in songbirds [23] we investigated the patterns of gene expression from song control regions (HVC, RA and Area X) in relation to the nucleus that governs the seasonal change in reproductive state, the POA. This approach facilitates the ability to dissociate the neurogenetic control of the production of song with those associated with the motivation to engage in song behavior. The genetic profiles in the song control regions and POA were analyzed in male starlings that had experienced photoperiodic regimens that resulted in different seasonal reproductive states. Furthermore, we gonadectomized starlings to evaluate the contribution of gonadal steroids for the photoinduced change in genetic profiles in both neural systems. Our analysis was designed to investigate broad variation in gene expression across these distinct brain regions.

## Methods

### Subjects

40 Male European starlings (*Sturnus vulgaris*) were collected in January 2007 from a local farm field site. Birds were group housed on the naturally occurring short days (11 L:13D) experienced at that latitude. All procedures were approved by the Institutional Animal Care and Use Committee at the Johns Hopkins University. Permission was granted by the farm owner to collect the starlings.

### Laparotomy and castration

Laparotomies were conducted under isoflurane (3-4% induction, then 1-2% maintenance) and the dimensions of the left testis were measured with calipers to the nearest tenth of a centimeter. Testis volume was determined using the equation V = 4/3π*a*^*2*^*b*, where *a* is half the width and *b* is half the length. Eight adult males were selected for castration, starlings were anesthetized and a small incision was made in the lower abdomen and the testes removed with forceps. For the sham surgeries thirty-two adult males were anesthetized and a small incision was made in the lower abdomen and the testes were touched with forceps but not removed. The castrated and intact males were housed on short day lengths to maintain sensitivity.

### Photoperiodic treatment

All birds were initially housed on short day lengths (11 L:13D) that maintained them in a state of photosensitivity. Birds were then transferred to long day lengths (16 L:8D) for either 7, 21 or 56 days (N = 8 for all groups; Figure 1B). The castrated starlings were placed on long day lengths for 7 days. One group remained on short days for the duration of the experiment in order to maintain a photosensitive state (N = 8). This photoperiod regime has been previously shown to induce the predicted physiological states [16,24]. The photoperiod manipulations were implemented such that brains were collected from all birds on the same calendar date between 1230 h and 1700 h. The time of sacrifice was counterbalanced across treatment groups. In order to account for changes in gene expression that could be attributable to singing [25-28], behavioral observations were taken on the final day between 1130 h and 1700 h. We did not observe any birds engaging in song during this time, therefore any changes in gene expression are most likely due to the photoperiodic and gonadal state of the birds. At the termination of the experiment, brains were removed and frozen in powdered dry ice and stored at −70°C. Body cavities were inspected to confirm castration and measure testicular volume.

### Brain tissue punches

Brains were sectioned using a cryostat (HM500 Carl Zeiss, Thornwood NY). Tissue sections were collected at the rostral extent of the brain and as each brain region of interest (ROI) was approached, tissue sections were stained for Nissl bodies to confirm the presence and boundaries of each nucleus. In brief, sections were directly mounted onto microscope slides and stained with thionin for two minutes, serially dehydrated in ethanol at 50%, 75%, 95%, 100% ethanol for fifteen seconds. The slides were then cleared in xylene (Fisher Scientific) then coverslipped with Permount (Fisher Scientific). Once ROIs were confirmed using a bright field light microscope (Zeiss Axioskop, Carl Zeiss, Thornwood NY), 120 μm tissue sections were then cut and ROIs were punched using 1.22 mm diameter plunges (Myneurolab, Leica, Richmond IL). Tissue punches were placed in 200 μl microfuge tubes and stored at −70°C until RNA extraction. For the POA, tissue punches were collected bilateral along the midline to the third ventricle from the caudal split in the tractoseptomesenphalicus to the rostral anterior commissure.

### RNA isolation and microarray hybridization

Tissues were sent to the University of Illinois, where all Songbird Neurogenomics Initiative microarray hybridizations are performed [23]. The standard protocol for Songbird microarray experiments is to use six replicates per treatment, therefore six of the eight birds were pseudo-randomly chosen and total RNA was extracted (Absolutely RNA Microprep kit, Stratagene), amplified (MessageAmp II aRNA Kit, Ambion), and Cy3/Cy5 dye-coupled (GE Life Sciences) as previously described [23]. RNA from each individual animal was hybridized to a single array. To enable cross-batch normalizations and analysis of the tissue, each array was hybridized with one experimental sample and a universal reference sample. The universal reference sample was a pooled composite of zebra finch (*Taeniopygia guttata*) brain mRNA, also amplified, that was hybridized on all of the SoNG microarrays [23]. The Cy3/Cy5 dye coupling was balanced (dye-flipped) between experimental and universal reference samples within each treatment group to control for potential dye incorporation and hybridization biases. The arrays were hybridized overnight at 42 degrees celsius in individual slide chambers (Corning), washed, scanned using Axon GenePix 4000B slide scanner (Molecular Devices) and visualized with GenePix Pro 6.0 (Molecular Devices) as described for all SoNG arrays [23]. Analyzed slide images were manually edited and aberrant spots (e.g., debris, scratches, or smears) were flagged for exclusion in downstream analysis. The validity of the cDNA zebra finch microarray used in the present study was established previously with RT-PCR and *in situ* hybridization [29,30]. All ESTs have been deposited in Genbank (Accession numbers DV944971 - DV962014, CK301200 - CK317559 and FE712085 - FE739917). Raw microarray data is archived and distributed in MIAME-compliant form using NIHs GEO (Gene Expression Omnibus) repository.

### Statistical analysis

One way ANOVAs were conducted on testicular volumes and Tukey’s pairwise comparison were used. For the arrays, data pre-processing and statistical analysis were done in R [31] using the limma package [32] from Bioconductor [33]. Spot intensity values were calculated using a standardized method for the SoNG arrays [29,30]; briefly, the median background was subtracted from the median foreground value (any negative/zero values were set to 0.5) [34], then a print-tip loess within-array normalization followed by a between-array scale normalization were performed [35]. A limma model equivalent to a 4 × 5 ANOVA (tissue x treatment) was used, and for each tissue, an F-test for treatment effect was calculated as well as a t-test between the castrated starlings and the intact starlings kept on long day lengths for 7 days. Because the False Discovery Rate correction [36] depends not only on a spot’s raw p-value, but how many other spots have low raw p-values, the same raw p-value in different tissues can have different FDR p-values. Therefore, to standardize the threshold for “significance” across tissues, a raw p-value cutoff of 0.001 was used to identify spots with a significant treatment effect within each tissue. At raw p-value < 0.001, the equivalent FDR level for overall treatment effect in each tissue ranged from 0.032 (HVC) to 0.082 (RA) to 0.096 (AreaX) to 0.142 (POA). Heatmaps of the significant spots for each tissue were made to assess the pattern of expression over the 5 treatments. Clusters of genes sharing the same pattern of expression (potentially co-regulated) in the heatmaps were determined by eye.

### Functional gene analysis

We performed an analysis of gene ontology (GO) to determine whether co-regulated gene sets were statistically associated with particular biological functions. We used Zebrafinch GO analysis ARK-Genomics ( http://bioinformatics.iah.ac.uk/tools/GOfinch) [37] that directly compares significant gene sets in HVC and POA against the complete microarray gene population. This analysis identifies which GO categories that are contained within the significant gene list occur more often than expected based on the distribution of GO categories of the whole microarray. We selected a p-value < 0.05 after hypergeometric analysis to analyze significantly up-regulated GO categories within a given gene set.

## Results and discussions

### Assessment of reproductive state

We first measured the change in testicular volume in order to confirm the starlings were in the appropriate reproductive conditions when the brains were collected. Figure 1C shows the testicular volumes across the four treatment groups (n = 8 for each condition). All males collected from breeding conditions (early breeding, LD7 and late breeding, LD21) had significantly greater volumes compared to non-breeding (LD56) starlings (*p* < 0.005). Furthermore, breeding birds had significantly greater volumes compared to pre-breeding birds (SD56; *p* < 0.001). These findings indicate that the starlings were collected while in the intended physiological states and provide a reliable indicator that the song system brain regions and POA exhibited the predicted changes in neural plasticity.

### Song system and POA gene expression profiles

**Figure 2:**
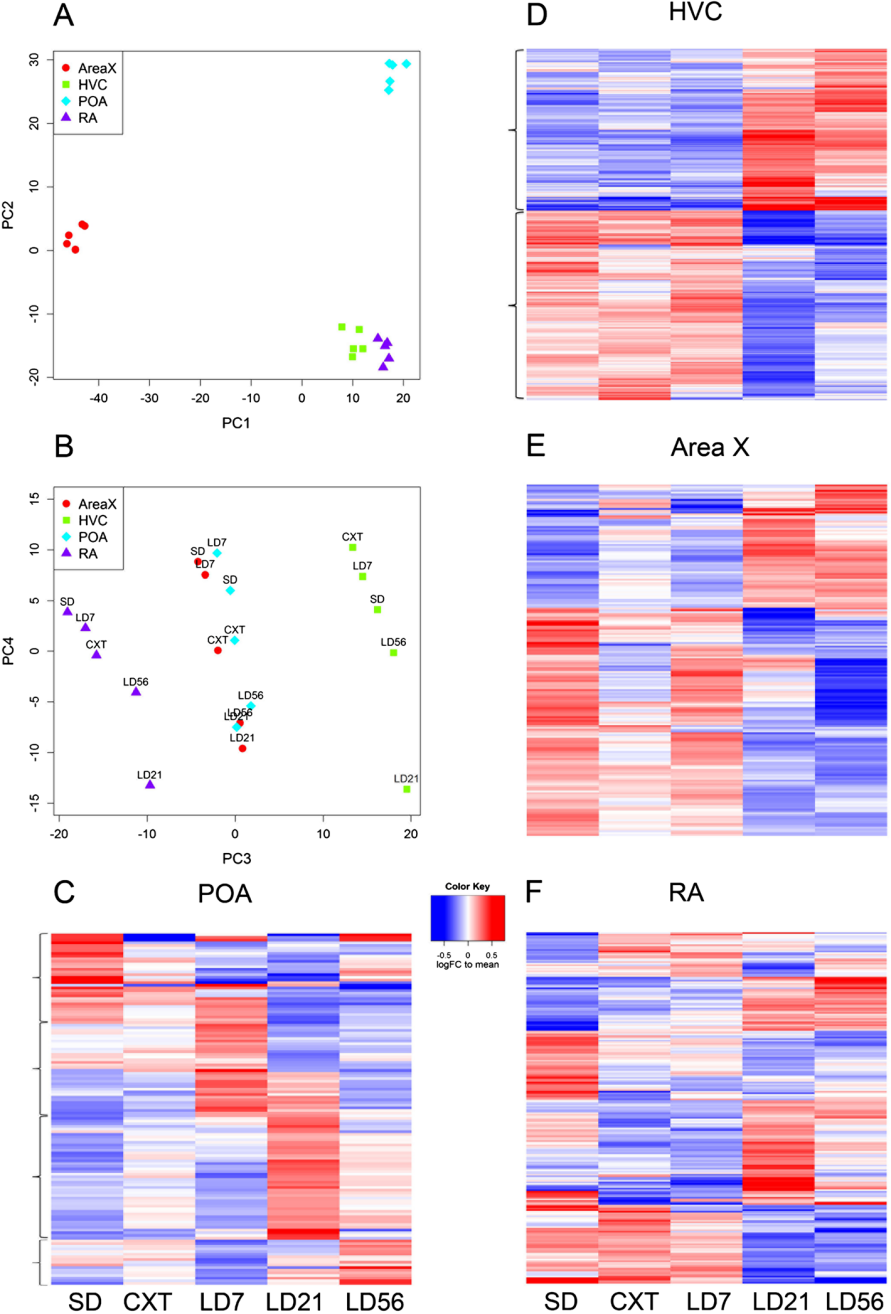
**Gene expression profiles from two brain systems that govern singing behavior.A**) PCA plot showing the separation of different brain regions on PC1 and PC2. **B**) PCA plot showing further separation of the brain regions on PC3 and some separation of the treatments on PC4. **C**). Heatmap of the 132 cDNAs in POA with significant changes across the treatments. The POA heatmap reveals waves of gene activation as starlings transition across the different photoperiodic states. **D**) Heatmap of the 590 significant cDNAs in HVC. This patterns shows a categorical shift between LD7 and LD21. **E**) Heatmap of the 194 significant cDNAs in Area X, also showing a categorical shift between LD7 and LD21. **F**) Heatmap of the 228 significant cDNAs in RA, which shows a mix of expression pattern types. Curly brackets on the left side of **C**) and **D**) indicate which groups of cDNAs were selected for GO analysis. Abbreviations: SD - short days for fifty-six days; CXT - castrated for seven long days; LD7 - intact for seven long days; LD21 - long days for twenty-one days; and LD56 - long days for fifty-six days.

**Figure 3:**
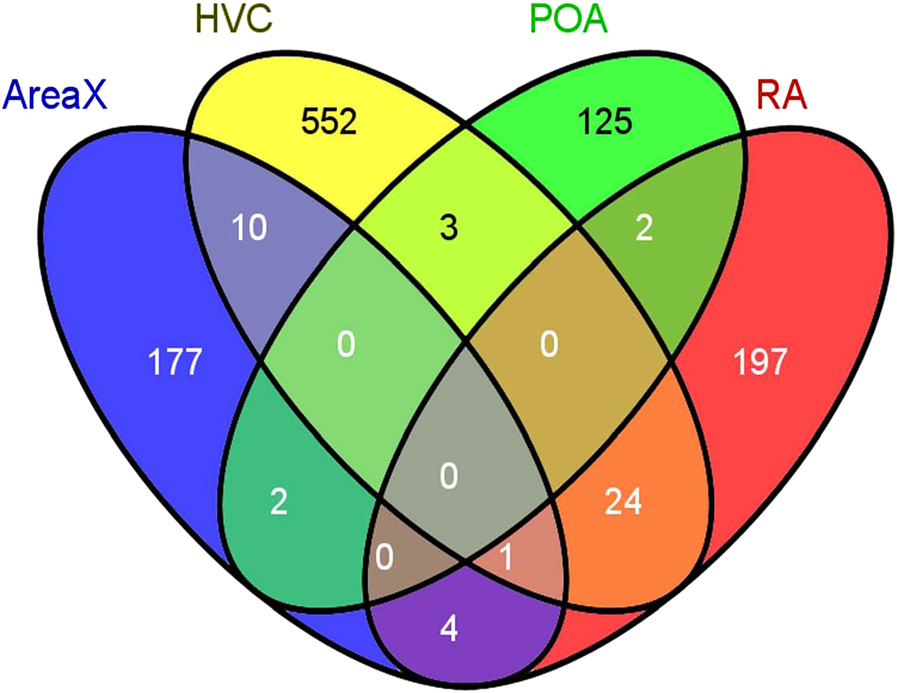
**Venn diagram illustrating the distribution of genes that exhibit significant variation across treatments for each brain region.** There were no genes observed to show a significant difference in expression patterns across all brain regions. This suggests that the variations in gene expression profiles are generally specific to the different brain regions. The greatest overlap in brain regions that show similar genes were found in HVC and RA (24 genes).

Next we measured gene expression profiles from the song control regions (HVC, RA, and Area X) and the POA from starlings. We used a spotted cDNA microarray with 20,160 addresses representing 17,214 non-redundant products of an estimated 11,500 genes designed specifically for songbirds [23]. Microarray hybridization data were analyzed with analysis of variance (ANOVA; Additional file 1: Table S1, Additional file 2: Table S2, Additional file 3: Table S3, Additional file 4: Table S4). The estimated values for each tissue x treatment group for all cDNAs were pulled from the model and principal components analysis were conducted to cluster the groups based on their overall expression levels (Figure 2). We found that PC1 separated Area X, whereas PC2 isolated POA (Figure 2A). PC3 separated HVC and RA, and PC4 illustrates the treatment group manipulation separation (Figure 2B). The pattern of nucleus separation due to the PCA analysis may be attributable similarities in gene expression due to the anatomical position. For example, the PCA analysis may have resulted in the separation of Area X due to the striatal/pallidal structure and the POA was separated next based on the hypothalamic structure. Finally, PC3 then separated out the two cortical structures: HVC and RA. For each tissue, we then generated a heatmap (Figure 2C-F) to visualize the overall changes in expression profiles for the cDNAs with significant within-tissue treatment effect (HVC 590, RA 228, Area X 194, and the POA 132). We then used Venny ( http://bioinfogp.cnb.csic.es/tools/venny/index.html) to evaluate the amount of genetic overlap within each brain region and observed remarkably low levels of similarity (Figure 3), suggesting the changes in expression observed are specific to the brain regions. One striking observation is that while Area X and HVC have very little overlap between significant gene lists, the heatmaps (Figures 2D and 2E) indicate that the pattern of expression across the treatments is very similar.

The overall pattern observed in the POA indicates a gradual transition across each reproductive state with marked changes in expression associated with different time points (Figure 2C). Specifically, as starlings transition from a pre-breeding state (i.e. SD) to a long day breeding state and subsequently long day non-breeding state (i.e. LD56) there are distinct changes in different cDNAs indicated by the clusters of up-regulated areas (i.e. red bands, Figure 2C). One particularly striking observation was the different patterns of cDNA expression between castrated and intact (LD7) starlings in the POA based on a visual examination, compared with almost identical cDNA expression in HVC. A T-test that tested mean differences between the CXT and LD7 groups revealed that 13/132 cDNAs (9.8%) in the POA were significantly different compared with only 10/590 cDNAs (1.7%) in HVC, 6/228 cDNAs (2.6%) in RA and 7/194 cDNAs (3.6%) in AreaX. Of the 13 changed cDNAs in the POA, only 2 were up-regulated in the castrates while 11 were down-regulated compared with intact LD7 males. These data are similar to those reported in another seasonally breeding species, the song sparrow (*Melospiza melodia*, 29). In this study, large groups of whole hypothalamic genes were shown to exhibit marked variation between breeding and non-breeding sparrows [29]. Although a comparison between our study and Mukai et al., 2009 [29] is limited due to anatomical specificity, it is intriguing that the broad changes in gene expression between breeding and non-breeding states revealed by the heat maps occur in two photoperiodic bird species.

When we compare the cDNA expression profiles of the POA to the song control regions, we observed a striking difference. The pattern of expression in HVC and Area X displayed a categorical switch during the transition from LD7 to LD21 (Figures 2D and 2E, respectively). During this period in starlings, like many seasonally breeding songbirds, the transition to a non-breeding state begins and the frequency and intensity of singing substantially decreases [38]. RA on the other hand exhibits different gene expression profiles across the reproductive states; some of the cDNAs follow the categorical switch between LD7 and LD21 but many others have already changed by LD7 and continue to change at LD21 and LD56. The absence of a similar pattern in RA compared to HVC and Area X may be attributable to the myotopic organization of this nucleus that is not shared by the other two nuclei [39,40]. RA has many functional subdivisions that project to the midbrain centers for vocalization, hypoglossal nucleus for the control of syringeal motorneurons, as well as connections with the brain stem nuclei involved in respiratory and laryngeal control (see 40 for a review). Thus, the gene expression profile in RA observed here may represent a plethora of changes involved in the variation in the motor production of calls and songs as well as those associated with respiratory and laryngeal coordination. These findings are of interest because it is often hypothesized that testosterone plays the primary role in driving the seasonal changes in neural substrates [21,22]. In this study we show that there is a low percent of changes in CXT compared to LD7 males in HVC (1.7%) and RA gene expression (2.6%). These data suggest, though, that the observed change in neuroplasticity and behavior regulated by the increase in testosterone requires the activation of fewer genes than might have been previously predicted.

### Functional analysis of gene expression

We selected cDNA sets from POA and HVC that included specific expression patterns as indicated by the curly brackets in Figure 2C and 2D. In order to analyze the “wave-like” patterns of expression in the POA, we selected distinct cDNA sets for SD, LD7, LD21 and LD56. Here, we use the term “wave-like” to describe the groups of genes that exhibit higher levels of expression across the photoinduced reproductive states (i.e. red bands in Figure 2C). Based on our selection, the GO analysis identified 132 significant functional categories (Additional file 5: Table S5). 56 categories were found for SD, 37 for LD7, 23 for LD21 and 16 for LD56 starlings and consisted of a number of functional categories (Table 1). For HVC, to analyze the “categorical” shift in cDNA expression, we grouped SD, CXT and LD7 treatments and compared the up-regulated cDNAs displayed in LD21 and LD56 starlings. We found that 90 functional categories were observed to be significant and were divided into 34 categories up-regulated in the SD, CXT, LD7 treatments compared to 56 categories in the LD21 and LD56 starlings (Additional file 6: Table S6). Several functional categories were found to be associated with the genetic “switch” in HVC across the reproductive states (Table 2). One particularly remarkable observation is the lack of similar functional categories in POA compared to HVC, which emphasizes the unique patterns of cDNA expression localized to distinct brain regions. Overall, these data are useful in identifying large suites of functional categories that underlie variation in brain function that is attributable to a highly significant social behavior.

**Table 1:** **Results of functional genomic analysis of major up-regulated gene expression sets in POA **(Figure 2**B)**

**GO Term**	**GO Description**	**SD**	**LD7**	**LD21**	**LD56**
GO:0004887	Thyroid hormone receptor activity	p<0.001			
GO:0003707	Steroid hormone receptor activity	p<0.005			
GO:0003708	Retinoic acid receptor activity	p<0.05			
GO:0005977	Glycogen metabolic process	p<0.05			
GO:0042826	Histone deacetylase binding		p<0.05		
GO:0016527	Brain-specific angiogenesis inhibitor activity		p<0.05		
GO:0017053	Transcriptional repressor complex			p<0.05	
GO:0004966	Galanin receptor activity				p<0.01
GO:0004945	Angiotensin type II receptor activity				p<0.05
GO:0008233	Peptidase activity				p<0.05

Gene ontology was used to determine significant functional categories that were associated with higher levels of gene expression across the photoinduced changes in the POA of SD, LD7, LD21 and LD56 starlings. Only significant values are shown (p<0.05) for candidate terms, see Additional file 5: Table S5 for full list.

**Table 2:** **Results of functional genomic analysis of major up-regulated gene expression sets in HVC **(Figure 2**C)**

**GO Term**	**GO Description**	**SD+CXT+LD7**	**LD21+LD56**
GO:0000226	Microtubule cytoskeleton organization	p<0.005	
GO:0060052	Neurofilament cytoskeleton organization	p<0.005	
GO:0006096	Glycolysis	p<0.01	
GO:0007264	Small GTPase mediated signal transduction	p<0.05	
GO:0030131	Clathrin adaptor complex	p<0.05	
GO:0000785	Chromatin		p<0.05
GO:0004707	MAP kinase activity		p<0.05

Gene ontology was used to determine significant functional categories that were associated with higher levels of gene expression in SD, CXT and LD7 compared to LD21 and LD56 starlings. Only significant values are shown (p < 0.05) for candidate terms, see Additional file 6: Table S6 for full list.

## Conclusions

This study has delineated a number of genes from the song control system and the POA that are expressed during the transition across seasonal reproductive states. We found that “waves” of gene expression consisting of a series of different genes and functional categories in the POA, are correlated with the physiological changes that are essential for the seasonal regulation for the motivation to engage in a highly social behavior (i.e. song). In addition, the POA is vital for regulating reproductive physiology and behavior in birds [41]. The data provided here significantly contribute to our understanding of the molecular changes associated with the onset and termination of reproductive states in songbirds. Moreover, discrete brain regions in the song system exhibit a pronounced switch in gene expression and subsequent functional categories that are specific to the individual song control nucleus. These findings suggest that vertebrate species that engage in highly complex social behaviors exhibit large changes in the activation of several genes across a number of different brain systems that are involved in regulating behavioral plasticity.

Previous microarray studies in songbirds have shown singing related changes in gene expression [25-28,42]. For example, in zebra finches there are a number of genes that are regulated by singing behavior in HVC, RA, Area X and the lateral magnocellular nucleus of the anterior nidopallium (lMAN; 28). Furthermore, there are marked developmental changes in gene expression within HVC [26] and the auditory forebrain [30] that may be involved in song learning. In this paper, we show photoperiodic and gonadal state changes in gene expression profiles that are independent of singing behavior. Direct comparison of genetic profiles obtained by different microarray formats is inherently limited. Yet, the present paper and Li and colleagues [26] found that serpin peptidase inhibitor (SERPINE), cytochrome c oxidase (Cox6c), microtubule-associated protein (MAP), mitogen-activated protein kinase (MAPK), exhibited variation in HVC expression levels. MAPK is critically involved in a range of cellular processes including: gene expression, differentiation, mitosis and apoptosis. Given that HVC incorporates new neurons throughout development and across seasons, it is possible that the lower levels of expression may play a pivotal role during the annual incorporation of new neurons.

It is well known that Gonadotropin-releasing hormone 1 (GnRH1) is involved in the photoperiodic regulation of seasonal reproduction in many songbirds, including starlings [16,43,44]. Photostimulated birds have high levels of GnRH1 mRNA expression and the onset of photorefractoriness is associated with a gradual decline to undetectable levels [43,44]. The patterns of gene expression identified in the current study provide other candidate systems that may be involved in regulating the photoperiodic control of reproduction. It was recently shown that the local production of thyroid hormones in the mediobasal hypothalamus permits the release of GnRH1 from the median eminence leading to the photoinduced gonadal recrudescence [45-47]. The observation of increased thyroid hormone receptor activity in starlings held on short days suggest that thyroid hormones may also underlie the termination of a photorefractory state and/or the maintenance of the photosensitive state. Furthermore, the identification of methyltransferase activity by the GO analysis suggests that epigenetic factors, specifically DNA methylation of the GnRH1 promoter, may be involved in the seasonal regulation of reproductive physiology and behavior.

Songbirds have emerged as a valuable animal model to study a number of biologically significant phenomena [48]. The recent sequencing of the zebra finch genome has greatly facilitated our ability to further our understanding of the relationships between genes, brain and behavior in a complex social organism [49]. The present study builds upon previous research that has established a connection between predictability in gene expression in the brain and changes in behavior in vertebrate and invertebrate species [2-4]. The observed changes in gene expression profiles across the brain regions occur on a yearly basis in starlings. The unique contribution of the present study is the demonstration that marked variability in gene expression profiles can be predictive of the different behavioral states. Here we show that discrete brain regions that are involved in regulating a social behavior show distinct genetic profiles that are not synchronized across the season. Future studies should investigate the precise relationship between the temporal changes in gene expression across disparate brain regions to contribute to the elucidation of the relationship between genes, brain and behavior.

## Competing interests

The authors have nothing to declare.

## Authors’ contributions

TJS and GFB participated in the design of the study. TJS conducted the experiment. KR performed the RNA isolation and microarray hybridization. TJS and JD analyzed the data. TJS, KR, JD, DFC, and GFB wrote the manuscript. All authors read and approved the final manuscript.

## Disclosure statement

The authors have nothing to disclose.

## Supplementary Material

Additional file 1 **Table S1.** Significant cDNA expression in HVC. Click here for file

Additional file 2 **Table S2.** Significant cDNA expression in Area X. Click here for file

Additional file 3 **Table S3.** Significant cDNA expression in RA. Click here for file

Additional file 4 **Table S4.** Significant cDNA expression in POA. Click here for file

Additional file 5 **Table S5.** Gene Ontology for significant biological pathways in POA. Click here for file

Additional file 6 **Table S6.** Gene Ontology for significant biological pathways in HVC. Click here for file
